# DASSL “Data Access Sharing Storage & Linkage” Proof-of-Concept: Health and Related Data Linkage in Ireland.

**DOI:** 10.23889/ijpds.v7i3.1908

**Published:** 2022-08-25

**Authors:** Orna Fennelly, Bruno Voisin, Marta Olszewska, Derek Corrigan, Frank Moriarty, Tom Fahey, Divyajyoti Sarkar, Sherif Nagy, Simon Wong

**Affiliations:** 1 Irish Centre for High End Computing; 2 Royal College of Surgeons

## Objectives

The DASSL Model, conceived by the Health Research Board, aims to overcome challenges to linking data in Ireland including lack of unique patient identifiers, inconsistent application of legislation and siloed data. The objective of the Proof-of-Concept is to develop and test a demonstrator technical infrastructure to support this model.

## Approach

A stakeholder committee of representatives from government, health services, data controllers, patients/public and researchers was established. National and international data sharing and linkage landscapes were reviewed via interviews, scientific papers and grey literature. Five case studies were developed to demonstrate different linkages, data and research purposes, with synthetic data mimicking real health/social datasets generated using data dictionaries, data controller input, national statistics and Synthpop, Synthetic Data Vault and General Adversarial Networks. The technical infrastructure remains under development with linkage software being evaluated. Stakeholders will test this infrastructure and a final report will outline considerations for a national solution.

## Results

Synthetic versions of administrative data, patient registries, electronic patient records, longitudinal cohorts, imaging and genomics are being generated. Matching variables and content data will be split and securely shared respectively with the Trusted Third Party (TTP) and Health Data Hub (HDH) on a project-by-project basis and at regular intervals. The selected linkage software will be used to match both unique identifiers and personally-identifiable data using probabilistic and deterministic techniques between datasets and with a population spine. The encrypted linkage key will be shared with the HDH, where the data view for each case study will be created by the Research Support Unit using the content data. A locked down virtual Safe Haven will support researcher access with disclosure control check on any outputs.

## Conclusion

A DASSL infrastructure could support sharing, linking and analysis of health and social data in Ireland. However, high quality data including matching variables need to be collected to produce beneficial findings. A national rollout requires a governance and legislative model, improvements in data collection, further public engagement and significant resourcing.

